# A dataset for splenomegaly and its related findings in CT imaging

**DOI:** 10.1016/j.dib.2025.112114

**Published:** 2025-09-27

**Authors:** Doaa Obeidat, Anas Bsoul, Malak Abdullah, Israa Jawarneh, Hassan Al-Balas, Mahmoud Ayesh

**Affiliations:** a Department of Computer Engineering, Jordan University of Science and Technology, P.O. Box 3030 Irbid 22110 Jordan; b Nanotechnology Institute, Jordan University of Science and Technology, P.O. Box 3030 Irbid 22110 Jordan; c Department of Computer Science, Jordan University of Science and Technology, P.O. Box 3030 Irbid 22110 Jordan; d Department of Internal Medicine, Jordan University of Science and Technology, P.O. Box 3030 Irbid 22110 Jordan; e Department of Diagnostic Radiology and Nuclear Medicine, Jordan University of Science and Technology, P.O. Box 3030 Irbid 22110 Jordan

**Keywords:** Medical image analysis, CT-scans, Splenomegaly, Classification, Diagnosis, Artificial intelligence, Deep learning

## Abstract

Splenomegaly, defined as an abnormal enlargement of the spleen, is a critical radiological finding associated with a spectrum of serious health conditions, particularly liver disorders and hematologic malignancies. While Computed Tomography (CT) scans are commonly utilized in clinical settings to detect and assess the severity of splenomegaly, recent studies have demonstrated its potential to reveal new insights into the underlying causes of splenomegaly, such as liver cirrhosis and lymphoma, when integrated with machine and deep learning models. This enables patients to receive timely and appropriate treatment, thereby reducing the risk of complications. However, research in this area remains in its early stages, primarily due to the limited availability of real-world datasets required to develop and train robust Artificial Intelligence (AI)-based models.

This article introduces a diverse dataset specifically curated for the study of splenomegaly and its associated findings. Collected at King Abdullah University Hospital in northern Jordan, the dataset includes 248 de-identified CT scans and patient data from 248 adult subjects (42% female), with a mean age of 48.2 years (ranging from 18 to 91 years). It encompasses cases both with and without splenomegaly, covering a range of diseases such as liver cirrhosis, lymphoma, leukemia, myeloproliferative neoplasms, and thalassemia. This work presents an open-access dataset dedicated to splenomegaly, aiming to address the lack of publicly available resources in this area. Furthermore, it supports the development of AI-driven diagnostic models for detecting splenomegaly, evaluating its severity, and identifying its potential causes. Additionally, it serves as an educational resource for medical students studying splenomegaly. The dataset is freely accessible via the Zenodo data repository, providing a valuable foundation for further research and advancements in diagnostic applications.

Specifications Table

**Table:** 

Subject	Computer Sciences / Medical Image Analysis
Specific subject area	Deep Learning; Machine Learning; Radiomics; Medical Imaging and Radiology; Computer-Aided Diagnosis; Splenomegaly; Hematologic and Liver Diseases Research
Type of data	Images (.dcm), Tables (.xslx). Raw.
Data collection	A total of 248 contrast-enhanced CT scans, encompassing cases with and without splenomegaly across various pathologies, were acquired between 2019 and 2023 at the radiology department of King Abdullah University Hospital. The imaging data, obtained in axial view using Brilliance 64-slice and Ingenuity 128-slice CT scanners, were retrospectively collected, anonymized, and exported via the RadiAnt Dicom Viewer. Additionally, patient data were extracted from medical records and radiological reports and validated under the supervision of specialized internal medicine physicians and radiologists.
Data source location	Institution: King Abdullah University Hospital City/Town/Region: Ramtha/Irbid Country: Jordan Latitude and longitude: 32.50167° N, 35.99417° E
Data accessibility	Repository name: Zenodo Data identification number: 10.5281/zenodo.15824373 Direct URL to data: https://doi.org/10.5281/zenodo.15824373
Related research article	None

## 1 Value of the Data

• The dataset addresses a critical gap in the study of splenomegaly-related findings by providing the first publicly available dataset that includes both splenomegaly and non-splenomegaly cases across various conditions. Its diversity significantly broadens its potential for research and diagnostic endeavors.
• The images in the dataset serve as a valuable resource for research in image processing, and computer vision. These annotated images enable researchers to develop and validate AI-driven algorithms in the area of liver diseases and hematologic disorders, where splenomegaly is a prominent radiological feature. Additionally, the dataset supports the analysis of cases without splenomegaly, aiding in the identification and comparison of distinctive patterns.
• The dataset is an important educational resource, offering medical students and professionals’ access to real-world data. By integrating patient data with CT images, the dataset provides hands-on experience in diagnosing and understanding splenomegaly and its associated conditions.
• The dataset offers multiple avenues for reuse across various research domains. First, the CT images, preserved in their original (.dcm) format, can be processed using both 2D and 3D deep learning techniques to extract and analyse relevant features, with the flexibility to be exported in different formats to support future analyses by other researchers. Second, researchers can leverage the dataset to train and test new deep learning models for disease diagnosis. Additionally, the classification annotations within the patient data provide a valuable resource for categorizing cases, enabling the development of AI-based models for detecting, assessing splenomegaly and identifying its underlying causes.

## 2 Background

Recent advances in AI technologies, driven by advances in hardware such as graphics processing units and the availability of datasets, have brought evolutionary changes in the health care sector [1]. The integration of AI-powered computer-aided diagnosis systems, particularly deep learning-based, into medical imaging workflows has played a vital role in automating medical image analysis and supporting clinical decision-making in a cost-effective and timely manner [2]. While numerous datasets exist for various medical image detection and classification tasks, such as skin cancer [3], brain hemorrhage [4], kidney tumours [5], and Covid-19 [6], there is a noticeable scarcity of datasets specifically dedicated to splenomegaly and its associated findings. Existing datasets, such as Meddeb et al. dataset [7] (72 lymphoma and 77 cirrhosis cases) and Cheng et al. dataset [8] (19 lymphoma, 120 benign cases), are privately restricted due to privacy concerns, limiting accessibility for broader research applications. To address this gap, we curated this dataset as a structured resource for research on splenomegaly diagnosis and related applications. Given the absence of a publicly available dataset for studying splenomegaly, this dataset aims to facilitate the development of AI-driven solutions in this field while also contributing to the enrichment and expansion of existing datasets for investigating other pathological conditions.

## 3 Data Description

### 3.1 Patient cohort

**Fig. 1:**
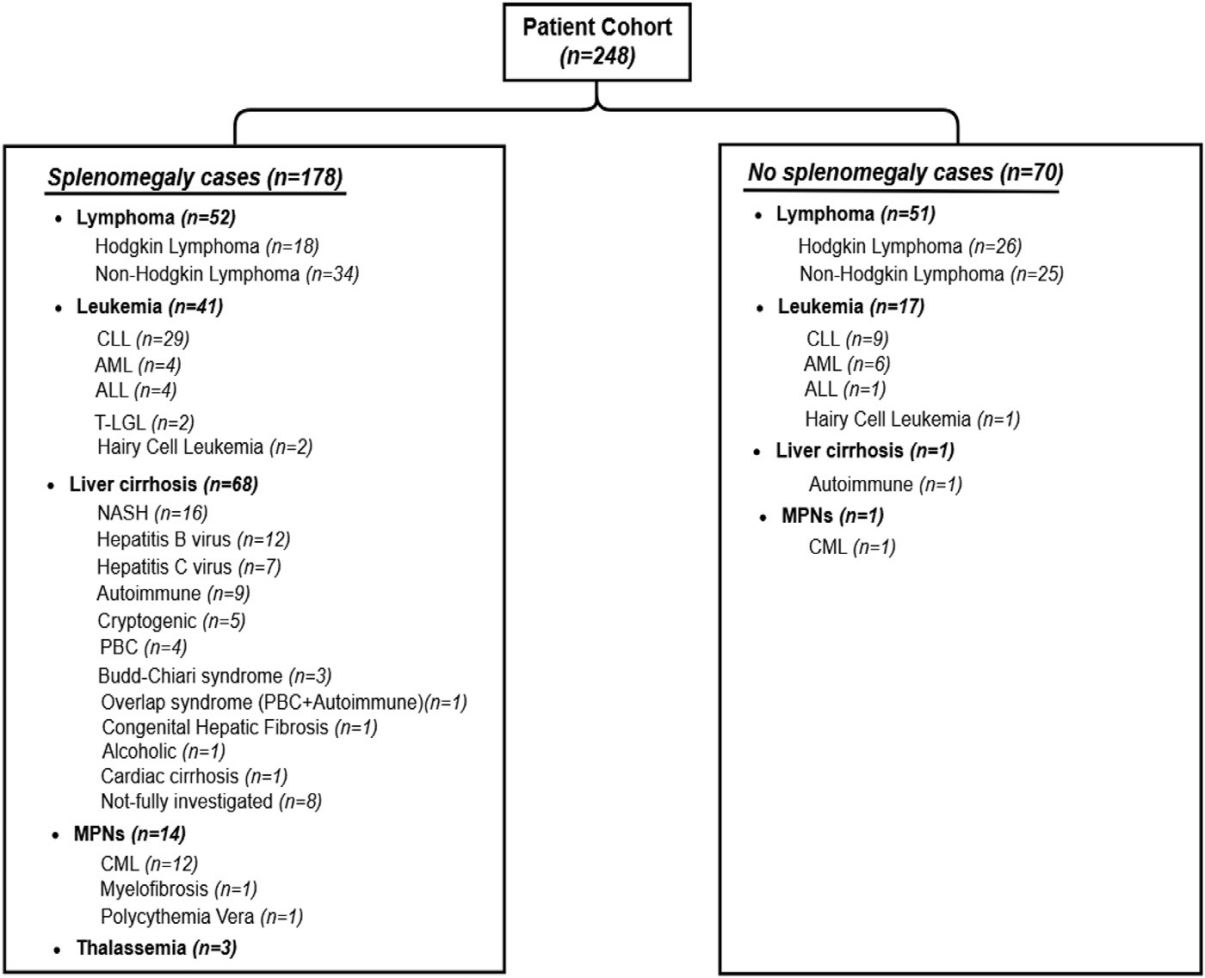
Categorization of patient cohort (*n* = 248) by splenomegaly status and diagnoses. Among the cases, 178 presented with splenomegaly and 70 did not, distributed across lymphoma, cirrhosis, leukemia, MPNs. Thalassemia was observed only in the splenomegaly group.

The dataset comprises CT scans and patient data from 248 adult subjects, including cases with and without splenomegaly. The cohort, 42 % are female, has a mean age of 48.2 years, standard deviation (SD) of 16.2, ranging from 18 to 91 years. The splenomegaly cohort includes 178 cases, with 38 % female subjects and a mean age of 49.03 years (SD = 15.5). This subset comprises 68 cases of liver cirrhosis, categorized as follows: hepatitis C virus (7 cases), hepatitis B virus (12 cases), autoimmune hepatitis (9 cases), non-alcoholic steatohepatitis (NASH) (16 cases), alcoholic cirrhosis (1 case), cryptogenic cirrhosis (5 cases), Budd-Chiari syndrome (3 cases), primary biliary cirrhosis (PBC) (4 cases), congenital hepatic fibrosis (1 case), overlap syndrome (PBC + autoimmune) (1 case), cardiac cirrhosis (1 case), and 8 cases without full investigation. The cohort also includes 52 cases of lymphoma, comprising 18 cases of Hodgkin lymphoma (predominantly classical Hodgkin lymphoma) and 34 cases of non-Hodgkin lymphoma (primarily diffuse large B-cell lymphoma, DLBL). Additionally, it contains 41 leukemia cases: 29 cases of chronic lymphocytic leukemia (CLL), 4 cases of acute myeloid leukemia (AML), 4 cases of acute lymphoblastic leukemia (ALL), 2 cases of T-cell large granular lymphocytic leukemia (T-LGL), and 2 cases of Hairy cell leukemia. The cohort further includes 14 cases of myeloproliferative neoplasms (MPNs) disorders, categorized as 12 cases of chronic myeloid leukemia (CML), 1 case of myelofibrosis, and 1 case of polycythemia vera. Additionally, this group includes 3 cases of thalassemia. The non-splenomegaly cohort comprises 70 cases, with 53 % female subjects and a mean age of 46.2 years (SD = 17.6). This group includes 51 lymphoma cases (26 Hodgkin and 25 non-Hodgkin, mainly DLBL), 17 leukemia cases (9 CLL, 6 AML, 1 Hairy cell leukemia, and 1 ALL), 1 case of liver cirrhosis (autoimmune), and 1 case of CML. The dataset distribution is illustrated in Fig. 1.

### 3.2 Dataset storage and annotation

The dataset is systematically organized into two primary directories for imaging data: one for splenomegaly cases and another for non-splenomegaly cases. Within each directory, subdirectories are structured according to specific conditions to facilitate classification tasks. Each patient's imaging files are stored in the corresponding condition-specific subdirectory. For example, a patient with splenomegaly due to lymphoma is placed in the lymphoma subdirectory within the splenomegaly main directory. Imaging files are then labeled at the volume level using a unique, customized identifier (Patient_ID) to ensure efficient access and management. Alongside the imaging data, a structured excel file (SplenoCT_data.xlsx) has been developed to organize key metadata, comprising two sheets: CT_Scans_Info and Patient_Data. The CT_Scans_Info sheet stores the technical attributes of CT images, including image resolution, where 95 % of images have a resolution of 512 × 512, slice thickness with 2 or 3 mm, and pixel spacing between 0.570312 and 0.9786184 mm. It also records the test time, indicating the year the image was acquired [2019–2023], the test area (abdomen pelvis, chest abdomen pelvis (CAP), and neck chest abdomen pelvis (NCAP)), and the number of slices, which varies between 156 and 887 slices per volume.

**Fig. 2:**
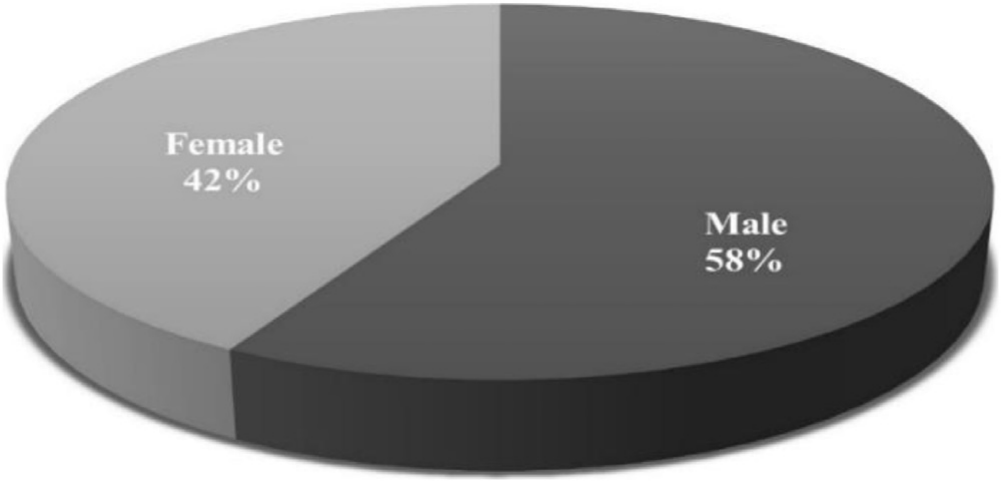
Distribution of the patient cohort by gender, showing 58 % males and 42 % females.
• The ``Age-Group” categorizes subjects into defined age groups: under 20, 20–30, 30–40, 40–50, 50–60, 60–70, 70–80, and over 80 (Fig. 3).

Similarly, the patient data was structured in another sheet (Patient_Data) containing patient-specific information and classification annotations. Each row corresponds to an individual patient, while the columns capture a range of clinical attributes. The patient data consists of eight attributes stored in text, numeric, and categorical formats, ensuring a comprehensive representation of each patient's medical profile. A detailed description of these attributes is provided below:

• The ``Patient_ID'' attribute assigns a unique identifier to each subject, following a structured naming convention: SPL or NSPL_Condition_Case [Number]. Here, SPL denotes the presence of splenomegaly, while NSPL indicates its absence. For instance, SPL_Cirrhosis_Case01 refers to the first case of cirrhosis with splenomegaly.
• The ``Gender'' attribute indicates whether the patient is female or male (Fig. 2).

**Fig. 3:**
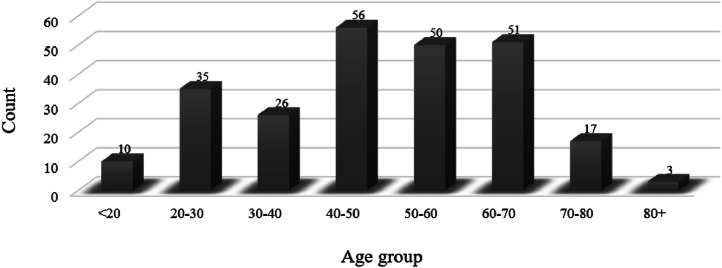
Age distribution of the patient cohort, categorized into eight age groups (<20 to >80 years). Case counts for each group are annotated above the corresponding bars.
• The “Type of Case” attribute classifies each case as either ``New'' or ``Follow-up.'' New cases represent initial diagnostic assessments, while follow-up cases involve monitoring disease progression, treatment effectiveness, or post-treatment outcomes (Fig. 4).

**Fig. 4:**
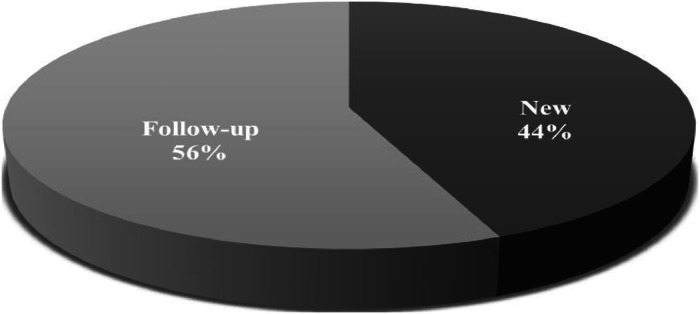
Distribution of the patient cohort by case type, showing 44 % newly diagnosed cases and 56 % follow-up cases.
• The ``Spleen_Maximum_Dimension'' attribute represents the longest measurable axis of the spleen in centimeters, extracted from CT scans. This measurement was taken from axial, coronal, or sagittal planes—whichever was the largest. It serves as a quantitative biomarker for detecting splenomegaly and assessing its severity.
• The ``Splenomegaly_Status'' attribute determines whether a patient has spleen enlargement based on predefined criteria. Assessing this is somewhat subjective, as there is no universally agreed-upon standard. For example, one study [9] classifies a craniocaudal spleen length of up to 12 cm as normal and values between 12 and 20 cm as indicative of splenomegaly. Other studies define splenomegaly using a craniocaudal length exceeding 9.5 cm and width greater than 10.6 cm on CT imaging [8,10]. In this dataset, the Spleen_Maximum_Dimension was adopted [11], applying a 14-cm threshold to identify cases of splenomegaly. This cut-off reflects the radiological assessment practices followed at King Abdullah University Hospital. Fig. 5 depicts the distribution of splenomegaly cases versus non-splenomegaly cases across genders.

**Fig. 5:**
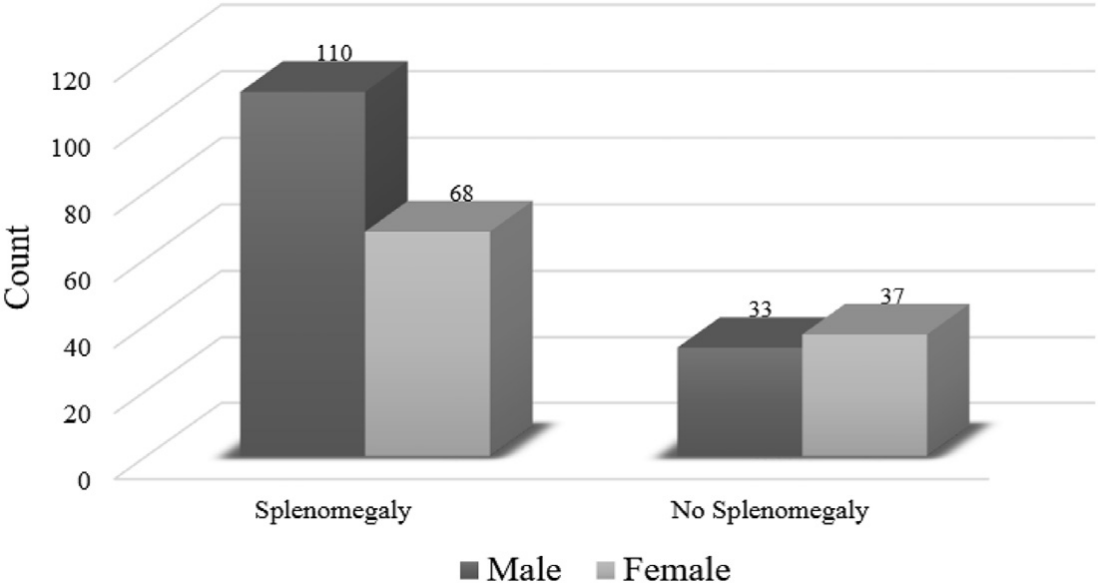
Gender-wise distribution of splenomegaly status. Male and female case counts are shown for both splenomegaly and non-splenomegaly groups, with counts displayed above each bar.
• The ``Diagnosis'' attribute classifies the primary conditions, regardless of the presence or absence of splenomegaly, into two main categories: liver cirrhosis and hematologic diseases, with the latter further divided into four subcategories: lymphoma, leukemia, MPNs, and thalassemia. Fig. 6 illustrates the distribution of hematologic diseases compared to liver cirrhosis, including cases both with a without splenomegaly, while Fig. 7 provides representative samples of CT scans across different pathologies, comprising both splenomegaly and non-splenomegaly cases.

**Fig. 6:**
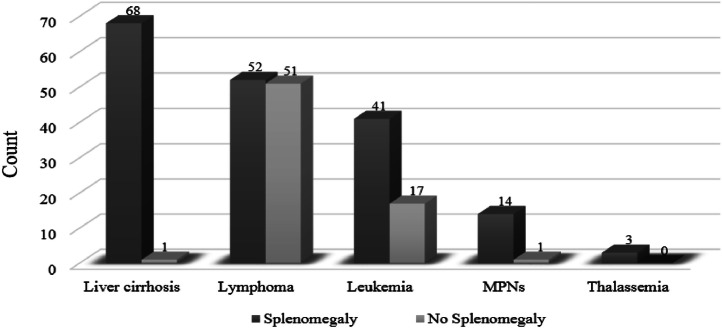
Distribution of patient diagnoses stratified by splenomegaly status. For each diagnosis (cirrhosis, lymphoma, leukemia, MPNs, and thalassemia), separate bars show the case counts with and without splenomegaly. Counts are displayed above each bar.

**Fig. 7:**
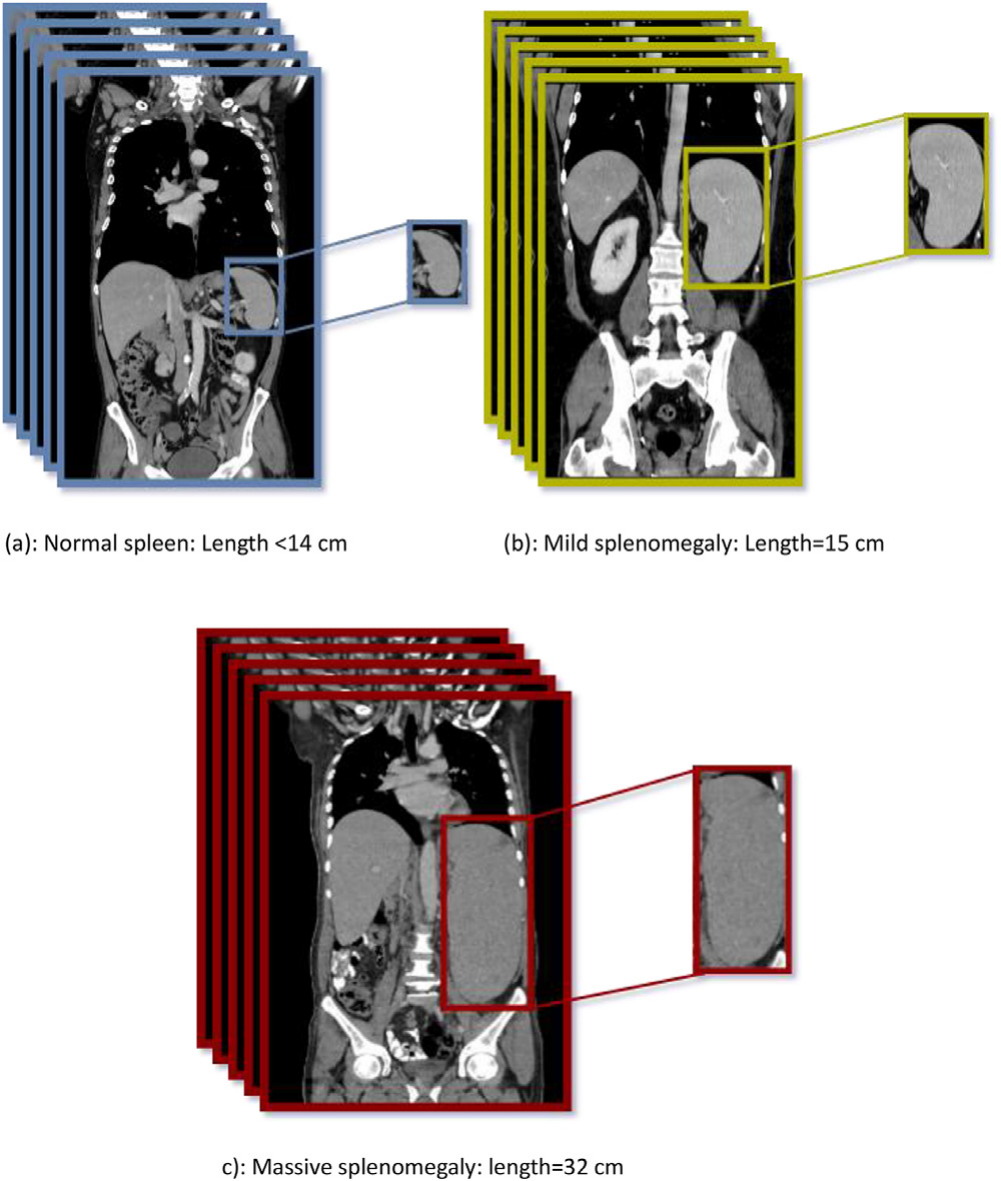
Samples of CT scans from our dataset (shown in the coronal plane) include: (a) a patient with a normal spleen length of less than 14 cm, diagnosed with leukemia; (b) a patient with mild splenomegaly measuring 15 cm, diagnosed with cirrhosis; and (c) a patient with massive splenomegaly measuring 32 cm, diagnosed with lymphoma.
• The ``Diagnosis_Subcategory'' attribute offers a more detailed classification of the main diagnosis ("Diagnosis" attribute) by dividing it into subcategories. For example, patients diagnosed with cirrhosis will have their specific type of cirrhosis identified, such as alcoholic cirrhosis, viral hepatitis cirrhosis, and others. Similarly, patients with leukemia will have their specific type of disease categorized, if applicable. Fig.1, as shown earlier, provides a detailed overview of these subcategories.

## 4 Experimental Design, Materials and Methods

A total of 248 cases, comprising patients with and without splenomegaly, were admitted to King Abdullah University Hospital between 2019 and 2023 based on specialist recommendations. These cases included newly diagnosed patients as well as follow-up cases, all with confirmed diagnoses of lymphoma, leukemia, liver cirrhosis, thalassemia, and MPNs, verified through histopathological analysis and specialized laboratory tests. To compile the dataset, electronic medical records and radiological reports were retrieved over an eight-month period to extract demographics (age, gender), spleen status, diagnosis details, and pathology reports. The initial selection consisted of 400 subjects; however, the dataset was refined by excluding cases with severe motion or breathing artifacts (33), unconfirmed diagnoses (21), missing radiological reports (3), non-contrast-enhanced scans (8), liver metastases (9), mixed diseases (4), and multiple CT images from the same subject (74). The final dataset (248 cases) was thoroughly reviewed and validated by physicians from the internal medicine department and radiologists to ensure accurate labeling and reliability.

As part of the diagnostic process, all patients underwent CT imaging to assess spleen status, identify abnormalities, evaluate organs involvement, and aid in disease staging. These scans were obtained through CT service interventions and meticulously reviewed and annotated by experienced radiologists. In cases where multiple scans were available for a patient, the radiologists carefully selected the single scan that best represented spleen involvement while maintaining diversity within the dataset. For the measurement of spleen dimensions across different planes, all assessments were performed by a single radiologist to ensure consistency and reduce interobserver variability. Two CT scanners were utilized: Brilliance CT (64 slice) and Ingenuity CT (128 slice) from Philips medical systems (595 Miner Rd, OH, 44143, United 26 States). These machines are equipped to provide high-resolution images. The contrast agents utilized included iopromide (Ultravist® 370 mg I/ml, Bayer, Leverkusen, Germany) and Omnipaque (Iohexol 350 mgI/ml, GE Healthcare, Cork, Ireland) with amounts varying between 100- and 120-ml.

Portal venous phase imaging was conducted 70–80 s after intravenous administration of the contrast agent. The CT images were provided in axial view through various imaging protocols, including abdomen pelvis, CAP, and NCAP in Digital Imaging and Communications in Medicine (DICOM) format, which is the standard for the digital storage and transmission of medical images. This format stores the voxel data intensities as well as metadata such as patient information, image dimensions, slice thicknesses, pixel spacing range, and others.

The DICOM files for each patient were systematically stored in dedicated folders, each labeled with the corresponding hospital ID to ensure an organized structure. To comply with anonymization guidelines, identifiable metadata, including patient names, IDs, ages, and birth dates, was removed using RadiAnt Dicom Viewer (RadiAnt DICOM Viewer). The anonymized images were then reviewed, exported at their original resolution, and assigned a new customized Patient_ID, as detailed in section Dataset Storage and Annotation. The baseline characteristic of the dataset is summarized in Table 1.

**Table 1:** Baseline characteristics of the dataset.

Characteristic	Details
Number of radiology centers	1
Number of unique individuals	248
Test-Time (Year)	2019–2023
Age range	18–91
Collection period	March 2023–October 2023
Type of cases	New, Follow-up (56 %)
Age: mean, SD (years)	48.2, 16.2
Gender ( %female)	42 %
Slice thickness range (mm)	2, 3
Number of slices per study (Min-Max)	156–887
2D image resolution (pixels)	95 % of images are in (512 × 512) resolution
Pixel spacing range (mm)	0.570312–0.9786184
CT Scans protocols (Test-Area)	Abdomen Pelvis, CAP, NCAP

## Limitations

The dataset has several limitations. First, the dataset is relatively small, consisting of 248 cases, and the data was collected from a single institution, which may introduce demographic and regional biases. Second, the dataset focuses on specific diseases causing splenomegaly, which may exclude rarer or atypical causes. Third, despite excluding low-quality CT scans, some scans may contain mild motion artifacts, streaks, or metallic noise due to respiratory motion and scanner-related factors. Finally, spleen measurements are subject to intraobserver variability, as minor differences in slice selection or measurement angle between assessments can lead to small, subcentimetric discrepancies.

## Ethics Statement

This single-institutional retrospective study was conducted according to the guidelines of the Declaration of Helsinki and approved by the Institutional Review Board (IRB) at King Abdullah University Hospital and Jordan University of Science and Technology, Jordan (Ref. 16/154/2023). The requirement for informed consent was waived due to the retrospective nature of the study. The personal data of patients were protected and anonymized in accordance with relevant guidelines. The researchers have the right to publish the data publicly.

## Credit Author Statement

**Doaa Obeidat:** Methodology, Software, Data curation, Formal analysis, Investigation, Validation, Writing – original draft, Visualization; **Anas Bsoul:** Conceptualization, Validation, Resources, Writing – review & editing, Supervision, Project administration, Funding acquisition; **Malak Abdullah:** Conceptualization, Validation, Resources, Writing – review & editing, Supervision, Project administration, Funding acquisition; **Israa Jawarneh:** Validation, Data curation; **Hassan Al Balas:** Methodology, Investigation, Resources, Supervision; Mahmoud Ayesh: Methodology, Investigation, Resources, Supervision.

## Data Availability

ZenodoSplenoCT_KAUH dataset (Original data).
